# Arrays of MicroLEDs and Astrocytes: Biological Amplifiers to Optogenetically Modulate Neuronal Networks Reducing Light Requirement

**DOI:** 10.1371/journal.pone.0108689

**Published:** 2014-09-29

**Authors:** Rolando Berlinguer-Palmini, Roberto Narducci, Kamyar Merhan, Arianna Dilaghi, Flavio Moroni, Alessio Masi, Tania Scartabelli, Elisa Landucci, Maria Sili, Antonio Schettini, Brian McGovern, Pleun Maskaant, Patrick Degenaar, Guido Mannaioni

**Affiliations:** 1 School of Electric and Electronic Engineering – Institute of Neuroscience, Newcastle University, Newcastle, United Kingdom; 2 Department of Neuroscience, Psychology, Drug Research and Child Health Section of Pharmacology and Toxicology, University of Florence, Florence, Italy; 3 Department of Health Science, Section of Clinical Pharmacology and Oncology, University of Florence, Florence, Italy; 4 Institute of Biomedical Engineering, Imperial College, London, United Kingdom; 5 Tyndall National Institute of Technology, Cork, Ireland; Zhejiang University School of Medicine, China

## Abstract

In the modern view of synaptic transmission, astrocytes are no longer confined to the role of merely supportive cells. Although they do not generate action potentials, they nonetheless exhibit electrical activity and can influence surrounding neurons through gliotransmitter release. In this work, we explored whether optogenetic activation of glial cells could act as an amplification mechanism to optical neural stimulation via gliotransmission to the neural network. We studied the modulation of gliotransmission by selective photo-activation of channelrhodopsin-2 (ChR2) and by means of a matrix of individually addressable super-bright microLEDs (μLEDs) with an excitation peak at 470 nm. We combined Ca^2+^ imaging techniques and concurrent patch-clamp electrophysiology to obtain subsequent glia/neural activity. First, we tested the μLEDs efficacy in stimulating ChR2-transfected astrocyte. ChR2-induced astrocytic current did not desensitize overtime, and was linearly increased and prolonged by increasing μLED irradiance in terms of intensity and surface illumination. Subsequently, ChR2 astrocytic stimulation by broad-field LED illumination with the same spectral profile, increased both glial cells and neuronal calcium transient frequency and sEPSCs suggesting that few ChR2-transfected astrocytes were able to excite surrounding not-ChR2-transfected astrocytes and neurons. Finally, by using the μLEDs array to selectively light stimulate ChR2 positive astrocytes we were able to increase the synaptic activity of single neurons surrounding it. In conclusion, ChR2-transfected astrocytes and μLEDs system were shown to be an amplifier of synaptic activity in mixed corticalneuronal and glial cells culture.

## Introduction

The traditional view of astrocytes is that their primary purpose is to provide biochemical support of the nerve cells, including trophic support, metabolic regulation, and regulating neurotransmitter concentrations in the synaptic cleft [1]. However, astrocytes also actively participate in synaptic transmission through gliotransmitter release [2], [3]. The understanding and the modulation of these processes could have particular translational impact to the pharmacology and neuroprosthesis communities as well as for neuro-computational studies. Fundamentally, indirect stimulation of astrocytes may lower operational power requirements of brain machine interfaces. Optogenetics is now a decade old genetic manipulation technique which can render nerve cells light sensitive [4]. The great advantages of the technique has been to provide genetically targeted excitatory [5] and inhibitory [6] control of neural circuitry with millisecond precision. The key issue for the neuroprosthesis community has been an intense light requirement of typically 10^15^–10^19^ photons/cm^2^ at 480 nm (instantaneous pulsed irradiance) [7], [8] which is close to the photochemical damage threshold of nerve cells [9], but also makes it challenging to create stimulation optoelectronics. High radiance optoelectronic arrays for specific use in retinal prosthesis have been previously developed [10]. However, inimplantable systems, local thermal dissipation becomes an increasing issue [11]. Therefore, we aimed to study ChR2-transfected astrocytes as a potential amplifier of neuronal signalling by means of increasing gliotransmission. For this reason, we wanted to explore the potential for optogenetically transfected astrocytes to influence the excitatory state of nerve cells, and thus bring down the threshold requirements for optoelectronic stimuli.

## Materials and Methods

### Ethical Statement

All animal manipulations were carried out according to the European Community guidelines for animal care (DL 116/92, application of the European Communities Council Directive 86/609/EEC). Formal approval to conduct the experiments described has been obtained from Italian Ministry of Health, according to DL 116/92. All efforts were made to minimize animal sufferings and to use only the number of animals necessary to produce reliable scientific data. No alternatives to animal experimentation are available for this type of experiments.

### μLED optoelectronic illuminator

Electronically drivenμLEDs were fabricated as part of the OptoNeuro FP7 project (www.optoneuro.eu) and transferred to the researchers on this project. These are fundamentally a micro-LED chip bonded to a CMOS (Complementary Metal Oxide Semiconductor) control chip. The array comprises of a 16×16 array of 20 µm diameter micro-emitters with a centre-to-centre pitch of 150 µm. The LEDs chip was fabricated from Gallium Nitride and the CMOS was fabricated from a standard 0.35 µm foundry process [12]. Bonding was achieved via flip-chip process, and the resulting die was packaged in ceramic pin grid array which was then placed on a PCB board and controlled by a PC via a MBED microcontroller.

### μLEDs controlling software

The CMOS driven optoelectronic array does not have a USB interface, so we have used a MBED microcontroller to act as an interface between a PC and the chip. This was programmed using the online software development kit from mbed.org. On the PC side, a software interface has been developed to provide intuitive functionality for the electrophysiology experiments. The software/hardware control can independently tune pulse widths of each of the micro-emitters down to 1 ms and is stable for many hours of recording. A hardware/software interface with standard patch-clamp electrophysiology software has also been developed via an in-house-designed trigger box for sending and receiving monitoring signals.

### Optical characterization

The emission spectrum of the optoelectronic array was measured by placing a USB2000 spectrometer (Ocean Optics) directly above the emitters. In order to measure radiance and efficiency, we used a Newport UV-818 calibrated photodiode and a Keithley Source Measure Unit 2612 (Keithley Instruments Inc.), with the diode placed just above the LED array. Variability in the emission power was tested by driving the μLEDs both individually and as part of a group. The on-sample emission powers were measured by placing the calibrated photodiode on the sample plane.

### Rat cortical cell cultures

Cultures of mixed cortical cells containing both neuronal and glial elements were prepared as previously described in detail [13] and used at 5–25 days in vitro (DIV). Pure neuronal cultures were prepared as previously described in detail [14] by seeding cortical cells (re-suspended in Neurobasal medium with B-27 supplement, GIBCO) onto poly-l-lysine-coated wells, used at 5–25 DIV. Either male and female animals were used.

### Plasmid amplification and cell transfection

Plasmid DNA encoding adeno-associated viral vector with light sensitive channelrhodopsin-2 under GFAP promoter (pAAV- GFP- hChR2 (H134R)-EYFP) or CatCh plasmid (pcDNA3.1(-)-chop2(1-309)[L132C]-EYFP) were purified using Plasmid Midi Kit (Qiagen) in according to the manufacturer's instruction. After 48 hours in culture at 70% confluence, astrocytes were transfected with 1 µg vector using Lipofectamine 2000 (Invitrogen) according to the manufacturer's manual. CatCh or ChR-2 expression in cortical neurons was achieved by electroporation (Lonza Biosciences Nucleofactor) using 2 µg of the construct.

### Electrophysiology

The recording chamber was mounted on an upright microscope (Nikon Eclipse E600FN) equipped with IR-DIC optics, 20× and 60× water-immersion objectives (NA = 1.00 and 0.8 respectively) and an IR-camera (Hamamatsu) for visually guided experiments. Flow rate was 1 ml/min and driven by gravity. Whole-cell recordings were performed at room temperature between 5 and 28 DIV. The intracellular solution contained (in mM) K^+^-gluconate (120), KCl (15), HEPES (10), EGTA (5), MgCl_2_, (2), Na_2_PhosphoCreatine (5), Na_2_GTP (0.3), MgATP (2), resulting in a resistance of 3–4 MΩ in the bath. The external medium contained NaCl (150), KCl (3), CaCl_2_ (2), MgCl_2_ (1), glucose (10) and HEPES (10); the pH was adjusted to 7.30. Clampfit v10.1 was used for offline analysis. No whole cell compensation was used. Signals were sampled at 10 kHz, low-pass filtered at 10 kHz, acquired with an Axon Multiclamp 700B and digitized with a Digidata 1440 A and Clampex 10 (Axon).

### Imaging of Fluo-3/Fura-2 fluorescence

Cultured cells were incubated in a solution containing (in mM): (150) NaCl, (10) Hepes, (3) KCl, (2) CaCl_2_, (1) MgCl_2_, (10) glucose (pH adjusted to 7.3) at 37°C for 30 min with the acetoxymethyl (AM) ester of fluo-3 and or fura-2 AM (5 µM, Molecular Probes). To aid solubilisation of fluo-3/fura-2 in aqueous medium, we added pluronic F-127 (1 mM, Molecular Probes). The dye was allowed to de-esterify for 30 min at room temperature. Coverslips containing fluo-3/fura-2-loaded cells were subsequently transferred to a continuously perfused microscope stage for imaging. Images were visualized with a 20× or 60× Fluor objectiveand acquired every 2 seconds. Exposure time was set to 200 ms and excitation was provided by a PE-1system (CoolLED) fitted with a 380±20 nm LED and a 470±30 nm LED. Fura-2 and fluo-3 fluorescence was recorded through (respectively) along pass filter (420 nm cut on) and aband pass filter (535±25 nm) with a Photometrics Coolsnap HP Camera set at −20°C. Fluorescence intensity was measured in cell bodies using Imaging Workbench 6 software (IndecBioSystem) and expressed as the ratio of (F−F0)/F0, where F0 is the baseline fluorescence intensity in cell bodies before any treatment. All measurements were corrected for the background fluorescence. Increases in fluorescence ratio greater than 0.1 were considered to be significant changes; baseline fluorescence values possessed a peak (F−F0)/F0 ratio of 0.01±0.01 on average. Experiments were performed at room temperature.

### Statistics

Pooled data throughout the paper are presented as mean ± standard error (SEM) of *n* independent experiments. Unless otherwise specified, statistical difference between means is assessed with a Student t-Test for paired samples (GraphPad Prism 5.0). When single recordings are shown they are intended to represent typical observations. Graphs, histograms and fittings were generated in GraphPad Prism 5.0. In Fig. S2 each response was normalised to a moving average of firing frequencies: average (all recordings) – average (preceding 4 readings and successive 4 readings).

## Results

**Figure 1:**
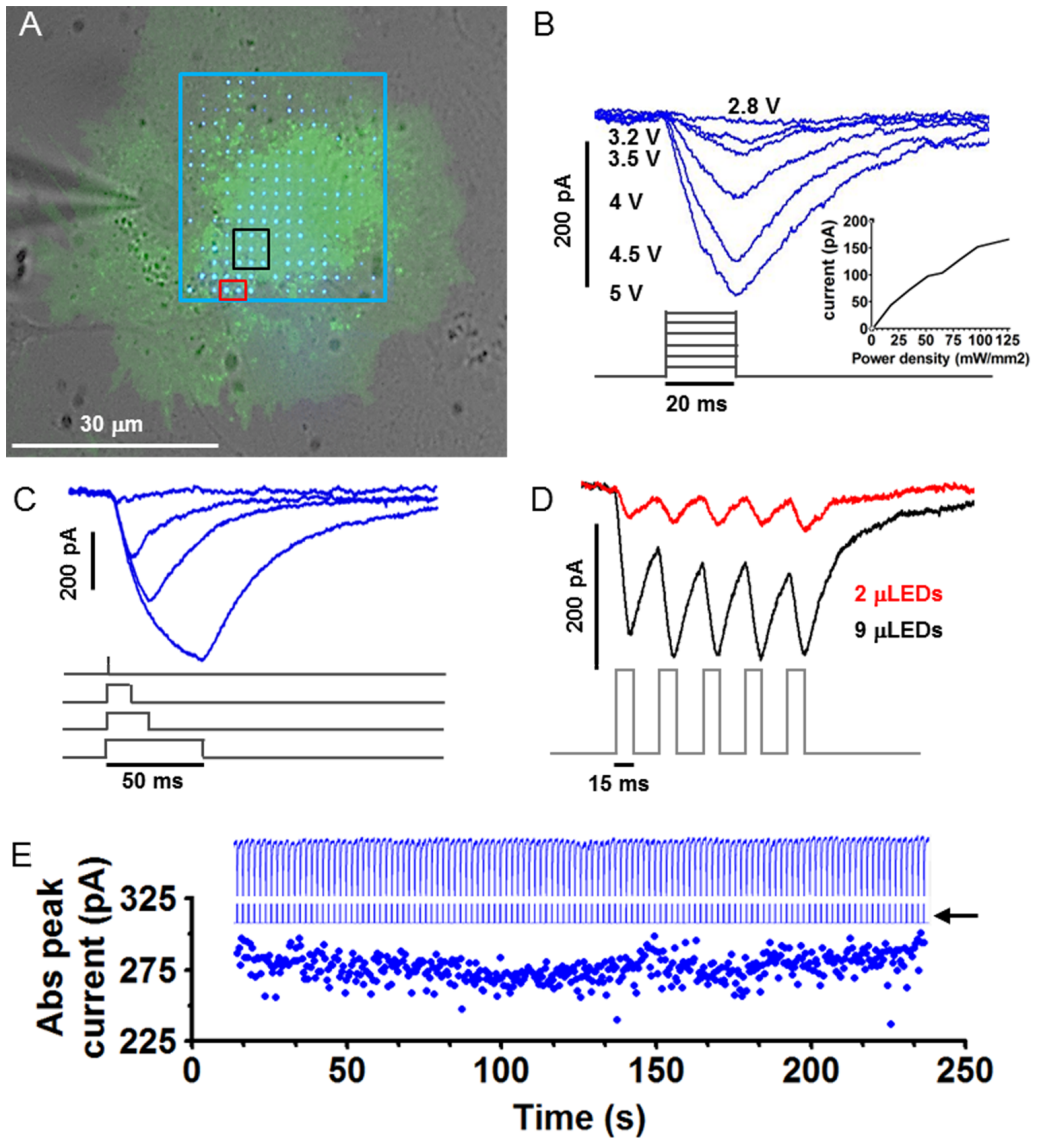
μLEDs finely modulate in time and space inward current in ChR2-transfected astrocytes. **A**,The ChR2+ astrocyte was stimulated with the whole matrix (blue box) or variable number of μLEDs (black and red boxes, 9 and 2 μLEDs, respectively) while recording the elicited inward currents in voltage clamp mode. Fine targeting and pulsing of the μLEDs on the cell was achieved overlaying in real time the fluorescent image to the μLEDs using a specific designed software. **B**, ChR-2 inward currents of different amplitude were recorded pulsing the whole matrix (blue box in **A**, pulse duration 20 ms) at different voltages (grey traces represent μLED stimulation pattern). Inset, mean inward current vs power density from different cells. **C**, μLEDs (blue box in **A**) can be finely modulated in time with submillisecond precision producing proportionally longer and larger ChR-2 currents (grey traces represent μLED stimulation pattern). **D**, Inward currents produced when 2 μLEDs (**A**, red box) or 9 μLEDs (**A**, black box) were pulsed 5 times at 33 Hz at different time on different locations (grey traces represent μLED stimulation pattern). **E**, The μLEDs irradiance is stable over time. When long term optogenetic light stimulation (central trace indicated by the black arrow, 200 ms pulse at 0.5 Hz, full led) is performed onChR-2 positive astrocyte the μLEDs produced stable current transients (Top trace) and peak inward currents (filled circles).

First, we tested the efficacy of the μLEDs array to elicit precise spatiotemporal current transients in ChR2-transfected glial cells. The μLEDs array was mounted on the microscope's camera port using a beam splitter allowing the μLEDs to be imagined onto the sample while observing it (Fig. 1A, S1). Whole cell patch clamp recordings were used to functionally verify the transfection and the capacity of μLEDs array to generate astrocytic ChR2-induced inward current. In each of the ChR2positive glial cells tested (n = 50), the μLEDs illumination produced inward currents using either the whole or partial array (Fig. 1A, whole array in white box and different number of μLEDs tested in coloured boxes). We could finely modulate ChR2-induced astrocytic inward currents by either modulating μLEDs power density (Fig. 1B and inset), pulse width of the illumination (Fig. 1C) and the number of illuminating μLEDs (Fig. 1D and 1A). We were also able to produce inward currents using pulses as short as 1 ms (Fig. 1C, red trace; power density = 34.66 nW/µm^2^@5V on cell, which equates to 34.66 pJ×pulse). In our previous paper we showed that the μLEDs irradiance is stable over time [15]. Here, we confirmed the array performance on a biological sample with long term light stimulation (200 ms pulse at 0.5 Hz, Fig. 1E, blue arrow) on ChR-2 positive astrocytes.The μLEDs produced stable current transients (Fig. 1E, black arrow) peaking at 275±20 pA (Fig. 1E bottom trace).

**Figure 2:**
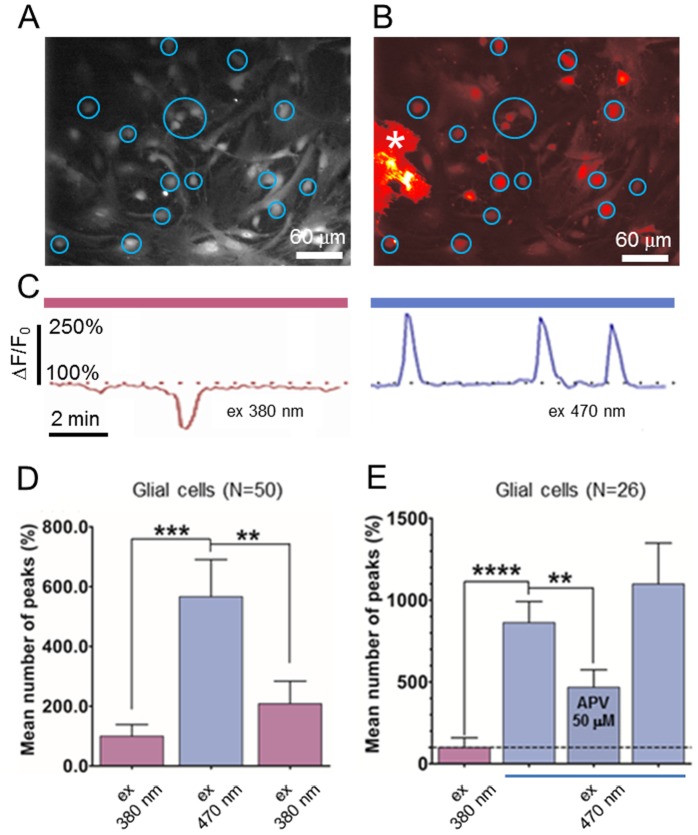
Stimulation ofChR2 positive astrocytesincreases glial cells calcium transients frequency. Cortical glial culture were co-incubated in fura-2-AM (**A**) and fluo-3-AM (**B**) and Ca^2+^ transients were monitored during UV [excitation (ex)380±20 nm] and blue light [excitation (ex) 470±20 nm] stimulation (200 ms light pulse @ 0.5 Hz; 10 min UV→10 min blue→10 min UV). The star (*) indicates the ChR-2 positive astrocyte. **C**, Time course of ChR-2 negative astrocyte during UV (left panel) and blue (right panel) illumination. Fura-2 downward peak indicates [Ca^2+^]_i_ increase, fluo-3 upward peak indicates [Ca^2+^]_i_ increase. **D**, Stimulation of the ChR-2 positive astrocyte with 470 nm light (blue column)increased calcium waves frequency to 566.7%±124.2% (UV vs Blue, paired t test p = 0.0002 – Blue vs UV, paired t test p = 0.0048). **E**,The increased Ca^2+^ waves frequency mediated by stimulation of ChR2 positive astrocyte was significantly reduced by APV 50 µM (UV vs Blue, paired t test p<0.0001 – Blue vs Blue+APV, paired t test p = 0.0019). Values are means ±SEM.

Then, we studied the optogenetic control of a glial network in culture via light stimulation of single ChR2 positive astrocytes in order to modulate surrounding ChR2 negative glial cells in pure astrocytic cultures by using calcium imaging as readout technique. For this experiment, we used a CoolLED PE system (see methods) to stimulate all the transfected cells in the area imaged by the objective. Since ChR2 is only partially stimulated at 380±20 nm (UV) [16], we used this wavelength as not-ratiometric Fura-2 exciting wavelength to assess the baseline activity of the culture (Fig. 2A and C, left panel).

We then compared this to the results obtained using Fluo-3/ChR2 peak exciting wavelength (blue light) (470±20 nm) to activate ChR2 positive astrocytes(Fig. 2B and C right panel) while recording the calcium activity of the surrounding ChR2-negative astrocytes (Fig. 2A and B, blue circles). Figure 2C shows a typical time course of a single ChR2 negative astrocyte excited at 380 and 470 nm, respectively. Stimulation at 470 nm increased calcium oscillation frequency in ChR2 negative astrocytes to 566.7±124.2% (p = 0.0002) over baseline activity (Fig. 2C, D and E) and this effect was reverted by switching back to 380 nm light (Fig. 2D) (208.1%±75.5% over the basal level; p = 0.0048). In another set of cells we aimed to pharmacologically block the ChR2-induced calcium wave frequency increase. Addition of NMDA selective antagonist D-2-Amino-5-phosphonopentanoic acid (APV 50 µM) during 470 nm light stimulation (but after the calcium wave frequency increase was established reaching 862±128.6% of the baseline level) reduced the induced increase to 469%±23.2%(p = 0.0019) of the baseline level (Fig. 2E). This partial block was reversible and calcium wave frequency re-increased to the pre-drug treatment level following APV wash out (Fig. 2E).

**Figure 3:**
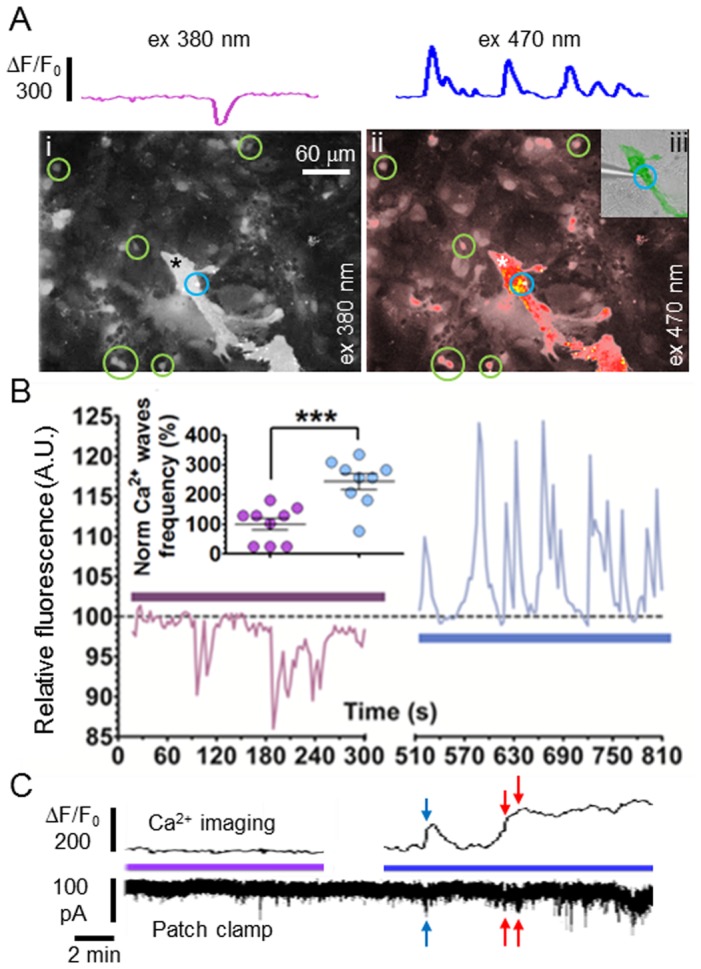
ChR2+ asctrocytic stimulation modulates neuronal calcium waves frequency. **A**, Bottom, snapshots from Ca^2+^ experiments during stimulation with 380 nm (**i**) and 470 nm (**ii**) light. Green circles indicate neurons, one of which (blue circle) was co-localizated with the ChR-2 positive astrocyte (star). Top, time course of one of the not colocalized neurons (circled in green). **B**,Time course of all circled neurons mean relative fluorescence and (inset)single cell measurement of calcium wave frequency (paired t test p<0.0001). **C**, Concurrent patch clamp and Ca^2+^imaging time course of the neuron circled in blue in **A**(**iii**). The red arrow shows the first wave (top) syncronised with the first sEPSCs burst and the red arrows show following sEPSCs bursts concomitant to internal calcium concentration increase.

After achieving optical modulation of glial cells network we then explored the interaction of optically modulated astrocytes and neurons. Initially, we stimulated ChR2 positive astrocytes by means of a CoolLED PE system while recording surrounding neuronal activity with calcium imaging technique (Fig. 3A). Figure 3B shows the time course of the mean calcium activity in 9 neurons during 380 and 470 nm stimulation (purple and blue), respectively.The modulatory effect on neuronal calcium wave frequency is shown in the inset and on a single cell in Fig. 3A (top).Moreover, in experiments where calcium imaging was coupled to concurrent patch clamp recordings (Fig. 3C), the increase in astrocytic intracellular Ca^2+^ concentration (blue and red arrows) was synchronized with spontaneous excitatory post synaptic currents (sEPSCs) burst of the colocalized neuron.

**Figure 4:**
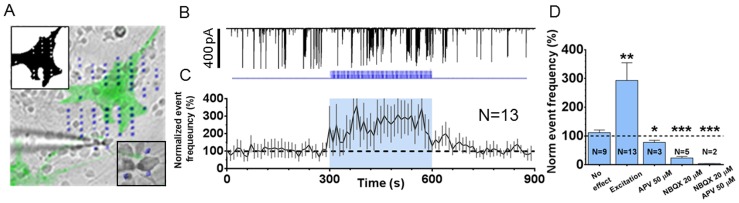
MicroLEDs-inducedChR2 positive astrocytes stimulation increases EPSCs frequency and is glutamate mediated. **A**, One of the ChR2 positive astrocyte in the field of view is light stimulated using 18 μLEDs (top left inset) while patch clamping from a nearby ChR2 negative neuron. Bottom right inset, a close-up of the ChR2-negative neuron showing that it is not illuminated by the μLEDs. **B**,Representative gap free patch clamp recording (black trace)performed on one of the 13 neurons that were modulated by the glial stimulationandstimulation pattern(blue trace)of the ChR2 positive astrocyte showing increase of synapticactivity following ChR2+ astrocytic light stimulation. **C**, Mean event frequency time course of the 13 neurons stimulated with the protocol as in **B** (blue trace) that showed a significant sEPSCs frequency increase over the baseline (black dashed line). **D**, The stimulation protocol was performed in 22 neurons, 13 of which showed a nearly 4-fold increase in the sEPSCs frequency. 9 out of the 22 neurons tested showed no significant sEPSCs frequency increase. Application of AMPA and NMDA receptor blockers after a significant increase of the sEPSCs frequency was established, reduced the latter to levels below the baseline level (all means paired t test vs control. No effect, p = 0.2635; Excitation, p = 0.0068; APV, p = 0.0371; NBQX, p = 0.0001; NBQX + APV, p = 0.0001.(Values are the means ± SEM).

To further characterize the optical gliotransmission, we used the μLEDs array to selectively light stimulate ChR2 positive astrocytes while recordings synaptic activity of a single neuron surrounding it (Fig. 4A). μLEDs light stimulated ChR2 positive astrocyte for 5 min using 200 ms long light pulses at 0.5 Hz (Fig. 4B blue trace). Simultaneously, we recorded the neuronal activity before, during and after the μLEDs induced stimulus. Figure 4B upper panel shows a typical sEPSCs timecourse following μLEDs stimulation of ChR2 positive astrocyte.

The average sEPSCs frequency activity increased to 295.5±53.4% following μLEDs stimulus selectively directed on ChR2 positive astrocyte (Fig. 4C,D and S2). Interestingly, sEPSCs amplitude did not change significantly (103.3±4% versus 109.1±15.2%, in control and during μLEDs induced stimulation of ChR2 positive astrocyte, respectively; n = 13).

The addition of the glutamate (NMDA and AMPA) antagonists, APV (50 µM) and NBQX (20 µM)to the bath solution during light stimulation and after the excitation was successfully triggered, significantly reduced the increased sEPSCs frequency to 78.20±4.0% and 23.2%±5.5% of the pre-light stimulation level (100%), respectively. APV and NBQX co-application almost abolished μLEDs ChR2-induced increase ofsEPSCs frequency (3.8%±1.0% of the baseline level) (Fig. 4D).

## Discussion

The data presented in this paper show that astrocytes can be finely tuned by ChR2 optogenetic stimulation and that the subsequent glutamate release rapidly affects the whole astrocytic network and the surrounding neurons. Perea and co-workers [17] have recently shown similar results in astrocytes of the primary visual cortex both for excitatory and inhibitory neurotransmission. The μLEDs system we previously tested in different cell lines [10], [18] is able not only to finely modulate ChR2 current in a single astrocyte but also to increase neuronal sEPSCs frequency in mixed cortical astrocytic/neuronal primary cultures.Following neuronal activity, the activation of astrocytes is mediated by neurotransmitter released from synaptic terminals [19], [20], [21]. The subsequent release of gliotransmitters from astrocytes has been reported to depend upon Gq GPCR activation leading to astrocytic type-2 IP3 receptor (IP3R2) activation and Ca^2+^ release from the endoplasmic reticulum [reviewed in [22]]. While this pathway has been implicated in gliotransmitter release, the precise mechanisms of gliotransmission remains debated [21], [23], [24], [2]. This is mainly due to our inability to selectively activate Ca^2+^ signals in astrocytes. Therefore, the exogenous generation of Ca^2+^ signals that mimic those evoked by neuronal stimuli should clarify the interactions between neurons and astrocytes and could finely modulate gliotransmission and the efficacy of neuroprosthetic devices.

For these reasons, we stimulated the astrocytes by means of ChR2-induced current showing that this direct astrocytic stimulation is cascaded onto the whole astrocytic network and increases neuronal spontaneous excitatory post synaptic current.

Interestingly, we also noticed that even if the currents elicited in Ca^2+^ translocating ChR2 (CatCh) positive astrocytes where on average 15 times larger than ChR2 (measured as area under the curve (AUC), Fig. S3) the neural network modulation was successfully achieved with ChR2, although previous reports suggest a better and stronger Ca^2+^ elevation by means of Ca^2+^-permeable light-gated glutamate receptor (LiGluR) [25] and CatCh [25], [26].

Recently, optogenetics elucidated the function of multiple neuronal circuits [27], [4], [28]. One of the most popular photo-switchable channel to activate neurons is the H314R channelrhodopsin 2 [ChR2(H134R)], a variant of the wild type ChR2 with reduced desensitization [29]. ChR2 is a cationic channel highly permeable to proton but weakly permeable to Ca^2+^ [30], [31]. In neurons, its photoactivation triggers Ca^2+^elevations which depend mainly on the secondary activation of voltage-gated Ca^2+^ channels (VGCC) [32], [33].In astrocytes, the photoactivation of ChR2 can trigger gliotransmitter release [34], [35], [36], [37], [17]. Indeed, in the rat brain stem retrotrapezoid nucleus, ChR2-expressing astrocytes reacted to long lasting (20–60 s) illumination by slow Ca^2+^ rises that lasted for minutes [35]. In the hippocampal CA1 region, blue light pulses induce rapid time-locked Ca^2+^ signals in astrocytes [37]. On the other end, mouse cortical astrocytes in culture showed a variable and weak Ca^2+^ elevations following ChR2 activation [25] while LiGluR and CatCh [7] evoked a reliable and robust Ca^2+^ signals in astrocytes [reviewed in [27] and [38]]. However, in our experimental conditions ChR2-transfected astrocytes showed a good efficacy in increasing [Ca^2+^]_i_ and in modulating glia to glia and glia to neurones transmission.

Unfortunately, due to the complexity of the astrocytic and neuronal network in cell cultures we could not discriminate the temporary resolution ofglial and neuronal cells stimulation. Pharmacological evidence showed that ChR2 non transfected astrocytes are partly stimulated through functional NMDA receptors activation which are present in cortical culture [39], [40]. However, since the increased Ca2+ waves frequency mediated by stimulation of ChR2 positive astrocytes was significantly but not completely reduced by APV 50 µM (figure 2E), we could not rule out other gliotransmitter release such as ATP through connexin channels (“hemichannels”) [41].

This study could have implications to the use of optogenetics for neuroprosthesis such as retinal prosthesis, visual brain prosthesis, brain and heart pacemakers. For practical application of optoelectronic prosthesis two platform technologies need to be optimized: 1) The biological expression – typically via viral vector of opsins with optimized biophysics. 2)The light generation and delivery mechanism to the optogenetically transfected cells [39].

In the case of the former, targeted delivery to specific cell types can allow for better communication and better sensitivity reducing the potential for long term photo-ionization damage [42]. In the case of the latter, a number of technologies are being developed including micro-light emitting diodes (μLEDs) [43] and optical delivery systems [44].

The μLEDs presented in this paper have delivered their light via microscope. If insulated, they could equally be placed against the tissue for similar effect. However, as neural tissue scatters blue light strongly, the individual addressability gets lost after a few hundred microns. Thus, either some form of light delivery system such as an optrode [43] would need to be incorporated or the chip would need to be shaped into a penetrating structure [45] to get closer to the target cells. It is also possible to place such LEDs directly against the tissue. However light scattering effects would mean they lose spatial resolution.

In the case of light emissive optoelectronics, there is a direct inverse correlation between efficiency and intensity. As such, creating mechanisms which reduce the light requirement will improve the efficiency and thus battery performance. As batteries in current neural pacemakers are largely non-rechargeable and need to last at least 5 years, this is an important consideration. Furthermore, for implants in the brain, inefficiency leads to thermal emission, which could cause undesirable heating of the neural tissue.

Currently the literature indicates that implantable devices should dissipate no more than ∼50 mW of thermal energy [11]. In this perspective, we demonstrated that ChR2transfection of astrocytes can be used to bring the requirement down in optogenetic systems, and this could have impact in future neuroprosthetic system design.

## Supporting Information

Figure S1 **System schematics.**
(DOCX)

Figure S2 A, Normalized moving average fit of the sEPSCs frequency time course. B, sEPSCs frequency during the relaxed and excited state and mean time (dA) to reach the excited state.
(DOCX)

Figure S3 **Example of current responses from a ChR2 positive astrocyte and a CatCh positive astrocyte elicited with a single 500 ms long pulse using the μLED array.**
(DOCX)
