# Supplementary material for: Arrays of MicroLEDs and Astrocytes: Biological Amplifiers to Optogenetically Modulate Neuronal Networks Reducing Light Requirement
Source: PLoS One. 2014 Sep 29;9(9):e108689. doi: 10.1371/journal.pone.0108689 (PMC4180921; doi:10.1371/journal.pone.0108689)
Supplement: Figure S1 — System schematics. (DOCX) [file pone.0108689.s001.docx]

**Figure S1**

**
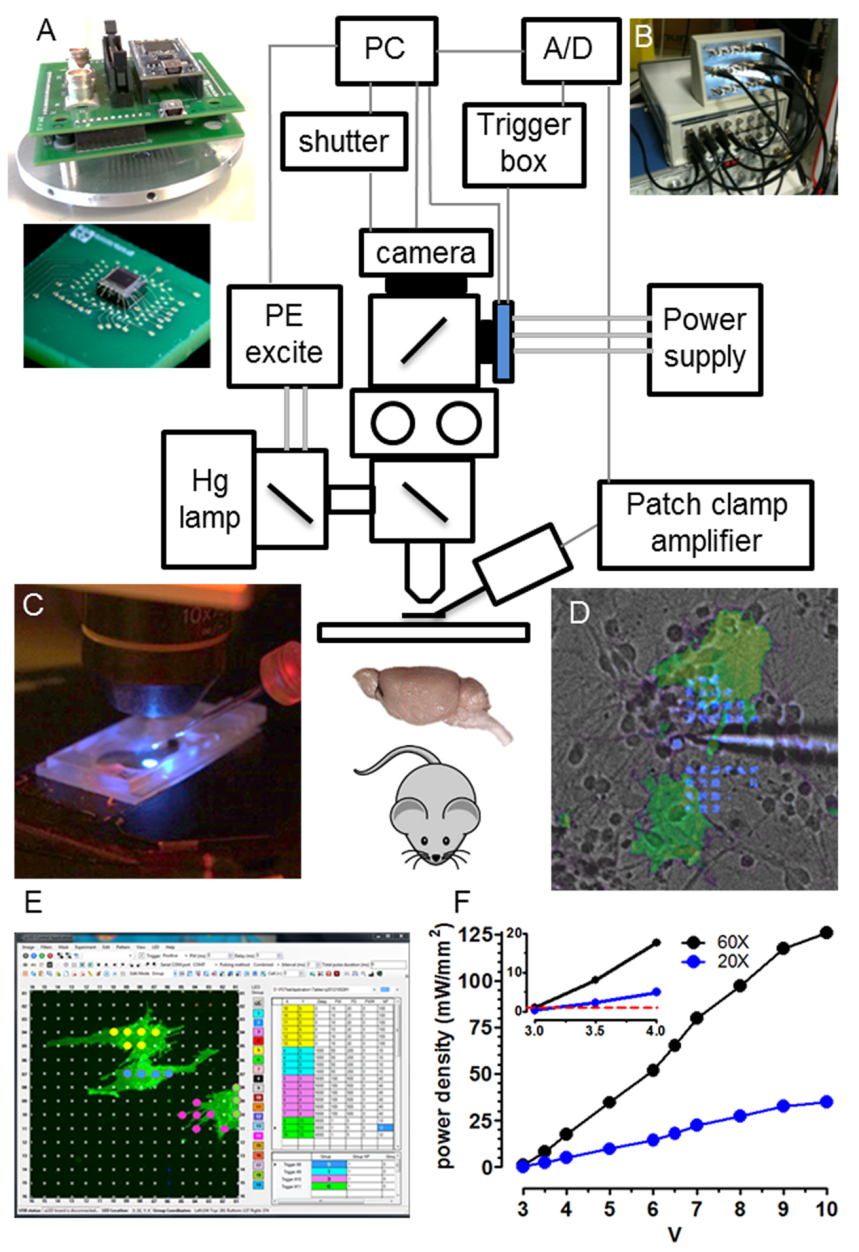
**

**Figure S1:** System schematics: the µLED array (bluebox on the schematics) was mounted on the microscope’s camera port using a beam splitter allowing the µLEDs to be imagined onto the sample while observing it. The µLEDs were connected to a PC (to control them), to a trigger box (to monitor the µLEDs signals via Clampex) and to two power lines (one for the µLEDs power and one for the CMOS controller). This arrangment allowed full opical stimulation control and targeting on the sample while performing patch clamp recording. The cell activity and the µLEDs pulse pattern were simultaneously recorded in clampex (as in fig. 4B). For Ca^2+^ imaging we also used either a Hg lamp or a Precise Excite system with a 380±20 nm and 470±20 nm macro LEDs. **A**) Top,the µLEDs array from the back where the microcontroller and the printed circuit board (PCB) are visible, **A** bottom, the 16x16 array mounted on a reduced size PCB (green board), **B**) The trigger box communicating with the A/D converter, **C**)µLEDs imaged onto a coverslip in the recording chamber, **D**) Patch clamp experiment stimulating the patched neuron and 2 different astrocytes from a mouse brain, **E**) Snapshot of the µLEDs controlling software overlaid in real time to ChR2+ astrocytes, allowing individual full control on the light stimulation and fine targeting. The fluorescent reporter GFP indicates the ChR2 expressing astrocyte (green), patched using a glass micropipette. Overlaid on the cells in **D** the µLEDs matrix in blue. **F**) Light intensity measurement taken with the two objectives (20x and 60x) used for the experiments. Calibration line in **A** bottom is 3 mm.
