# Supplementary material for: Arrays of MicroLEDs and Astrocytes: Biological Amplifiers to Optogenetically Modulate Neuronal Networks Reducing Light Requirement
Source: PLoS One. 2014 Sep 29;9(9):e108689. doi: 10.1371/journal.pone.0108689 (PMC4180921; doi:10.1371/journal.pone.0108689)
Supplement: Figure S2 — A, Normalized moving average fit of the sEPSCs frequency time course. B, sEPSCs frequency during the relaxed and excited state and mean time (dA) to reach the excited state. (DOCX) [file pone.0108689.s002.docx]

**Figure S2**


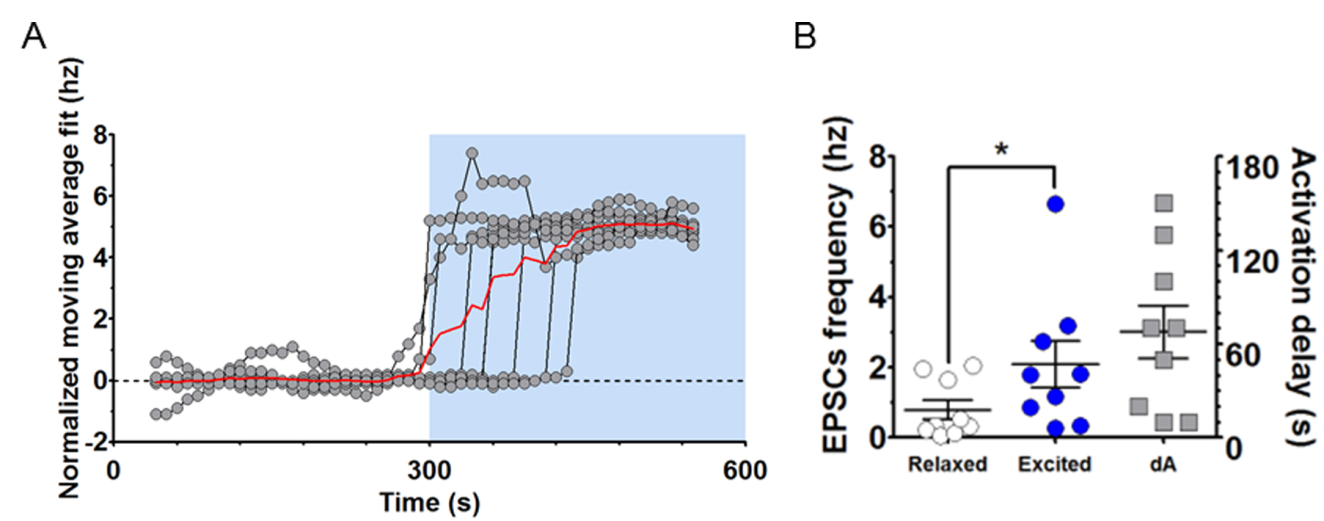


**Figure S2**: **A**) Normalized moving average fit (mean fit superimposed in red) of the sEPSCs frequency time course of 9 neurons receiving 200 ms µLEDs pulsed light stimulation for 5 min at 0.5 Hz starting from T=300 s. Each response was normalised to a moving average of firing frequencies: average(all recordings) – average(preceding 4 readings and successive 4 readings).
**B**) sEPSCs frequency during the relaxed and excited state and mean time (dA) to reach the excited state for the 9 neurons plotted (Relaxed vs excited, paired t test p=0.0191).
