# Supplementary material for: Arrays of MicroLEDs and Astrocytes: Biological Amplifiers to Optogenetically Modulate Neuronal Networks Reducing Light Requirement
Source: PLoS One. 2014 Sep 29;9(9):e108689. doi: 10.1371/journal.pone.0108689 (PMC4180921; doi:10.1371/journal.pone.0108689)
Supplement: Figure S3 — Example of current responses from a ChR2 positive astrocyte and a CatCh positive astrocyte elicited with a single 500 ms long pulse using the μLED array. (DOCX) [file pone.0108689.s003.docx]

**Figure S3**


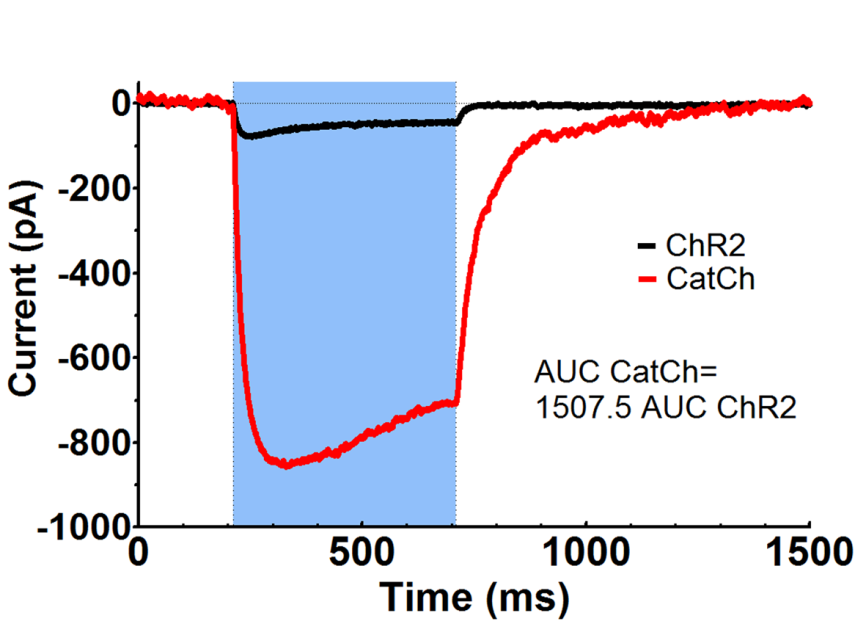


**Figure S3**: Example of current responses from a ChR2 positive astrocyte (black trace) and a CatCh positive astrocyte (red trace) elicited with a single 500 ms long pulse (blue shaded area) using the μLED array. The inward current elicited (measured as Area Under the Curve from the pulse start to the trace returning to the baseline level) was typically in the range of 15 times larger for CatCh positive astrocytes.
